# Supplementary material for: Identity leadership and adherence to COVID-19 safety guidance in hospital settings
Source: PLoS One. 2024 Jan 19;19(1):e0293002. doi: 10.1371/journal.pone.0293002 (PMC10798463; doi:10.1371/journal.pone.0293002)
Supplement: S1 File — (DOCX) [file pone.0293002.s001.docx]

**Supplementary materials**

**Full Interview Schedule**

**Introductory paragraph**

“Thank you for taking the time to meet with me today. As you know this is part of a project exploring staff-to-staff transmission of Covid-19. Today we would like to talk to you about your experience of the Covid-19 pandemic, specifically discussing safety guidance, how the guidance has been communicated to you and how your relationship with co-workers and management affects how you view safety guidance.”

**Knowledge**

1. Are there specific times/areas in your department where you think there might be particularly high risk of staff-to-staff covid-19 transmission?

**Guidance& Communication**

1. How are the Covid-19 safety guidelines communicated to everyone in your department? (Prompt: Do you feel that this is adequate, or can you think of ways in which this can be improved in any way, for example, might there be additional helpful ways, what have you found effective/ineffective?)
2. How regularly are you provided with updates about the guidance? (Prompt: Do you feel that this is adequate, or can you think of ways in which this can be improved in any way?)
3. Who is it that communicates Covid-19 safety guidance and updates to staff? (Prompt: Is the guidance communicated by someone who works on the same ward and/or of the same profession?)
4. And what is your relationship like with [this person] that gives you the Covid-19 safety guidance?
5. How clear/unclear is the Covid-19 guidance for hospital staff? (For example, is it clear when you should or should not be wearing different types of PPE like face masks, gloves, face shields?)
6. What are the conversations between management and staff about Covid-19 like? (Prompt: For example, do they feel like it’s a two-way conversation?)
7. What adjustments have been made to how guidance is communicated throughout the pandemic?

- Thinking about how the Covid-19 safety guidance was communicated, which methods do you find work best?

1. Thinking about how the Covid-19 safety guidance is communicated, do you feel that the guidance provided to staff is consistent between communicators, for example, are those providing guidance providing the same guidance to everyone or are there inconsistent parts?
   - If not – How does this make you feel about adhering to the Covid-19 safety guidelines? Can you think of ways that communication could be made clearer?
2. To what extent do you feel confident that you know and understand the Covid-19 safety guidelines?

- Are there ways in which, for example, the information can be communicated more clearly? (Prompt: How would you like to be informed about guidance updates and why?)

1. To what extent do you trust the Covid-19 safety guidance? (Prompt: What makes you say that? What aspects of the guidance make you feel safe in your work environment?)

**Guidance in practice**

“We are interested in exploring how the Covid-19 safety guidance works in practice and so the following few questions relate to your experiencing of adhering to the guidance on the front line”

1. How easy or difficult do you find it is to follow the Covid-19 safety guidelines? (Prompt: Can you give some examples? Why do you think that might be?)
2. How has your physical work environment been adapted to follow COVID-19 guidance?
3. How have the changes to your physical work environment impacted your day-to-day working practices? (Prompt: changes to office spaces, use of computers, handovers/ward meetings).
4. How do you find adhering to the physical distancing guidelines when you’re with colleagues say for example during breaks, handovers? (Prompt: what about in different environments, e.g., while onwards?
5. In what ways would following the Covid-19 guidelines be made easier? Prompt: Aspects relating to resources, or communication for example)
6. What motivates you to follow the Covid-19 safety guidelines?

**Group definition and dynamics**

1. In what ways has covid-19 impacted the dynamic among staff in your area? (Prompt: in what ways do you think it has impacted your work as a team?)
2. What would a typical staff member do in a situation where a colleague was upset? (Prompt: And how would they have acted if a colleague was upset before the pandemic?)
3. To what extent do you feel that members of your team follow the Covid-19 safety guidance? (Prompt: Why do think that might be?)
4. How would you feel if you saw a work colleague not following the Covid-19 safety guidance and how would you respond?
   1. How do you think others would respond?
   2. How would you feel if the person not following the guidance was in a different role from you, e.g., your line manager?
5. Having been a member of staff working through the pandemic, how do you feel your role has changed?
6. What is the working relationship like between staff and line managers in your department?’ (Prompt: do you feel you’re in it together/part of the same team? Or with senior managerial staff in the hospital?)
   1. If positive, what do you believe contributes to this?
   2. If negative, what do you believe contributes to this?

**Closing**

1. That’s all the questions I wanted to ask. Is there anything you want to ask that we haven’t covered?

Thank you for taking the time to share your experiences. I have really enjoyed listening to your experience and views on everything. We will be conducting interviews over the next few weeks but will be providing regular updates to the staff advisory groups and you should hear about the progress of the project. If you have anything you would like to add please feel free to get in touch.
