# Supplementary material for: Identity leadership and adherence to COVID-19 safety guidance in hospital settings
Source: PLoS One. 2024 Jan 19;19(1):e0293002. doi: 10.1371/journal.pone.0293002 (PMC10798463; doi:10.1371/journal.pone.0293002)
Supplement: Attachment — Submitted filename: Response to reviewers.docx [file pone.0293002.s002.docx]

Dear Editor and reviewers,

Thank you for the time you have taken to provide us with your valuable feedback. The comments were insightful and have substantially improved our manuscript. We have made multiple changes following your feedback which we list below.

**Introduction**

**Reviewer 1 requested more information on “how the theory (social identity view of leadership) allows for personal identity can affect group behaviour – as this is surely not only restricted to social identity”.**

We thank the reviewer for this fair point. However, we feel that focusing on personal identities is beyond the scope of this particular paper. The interviews focused on team relations and adherence to the guidance. At times, personal factors such as not wanting to take disease home to loved ones came up, but these were not related to our main research questions on views of identity leadership.

**Reviewer 1 requested “a small elaboration on the four dimensions of Steffens et al’s framework” to “better understand why two dimensions were considered relevant and the other two not”.**

We have expanded this description to explain the four dimensions of Steffens et al’s model to more clearly demonstrate why we selected two dimensions (prototypicality and advancement) as most relevant to explore (see lines 101-107). In this section, we also clarified how the early conversations with NHS staff highlighted the relevance of these two dimensions.

**Reviewer 1 requested clarity on “who was being approached in this study as constituting the group”.**

The group is subjective, and the participants themselves brought up who was relevant for their group. We appreciate that this was not clear so we have added a clarification within the manuscript when discussing the interview schedule in the methods section that staff were able to create their own categorisation of who was part of their group/team (see lines 218-220).

**Reviewer 2 made suggestions about specific line numbers which we addressed:**

**p. 2 line 65: Is ‘demonstrates’ the correct word here?**

We have changed ‘demonstrates’ to ‘proposes’ (see line 66).
 
**p. 3 line 86: Is ‘conceives’ the right word here?** 

We have changed ‘conceives’ to ‘highlights’ (see line 87).

**p. 3 line 90: I’m not sure what ‘achieving influence at the group’ means. Against the group?**

Achieving influence at the group is not necessarily against the group but not within the best interests of the group (e.g., by engaging in more authoritarian styles of leadership which do not engage through the group but instead try to guide change by directing at those they are trying to lead).
 
**p. 3 line 103: Is this OSF site the date for the present study or a different one? It’s not clear from the wording. I’m confused by the status of the ‘conversations’ mentioned here.”**

The OSF site is a project for the present study, where the final report from the broader project has also been made available for this paper. These initial conversations were part of the broader project before the interviews that provides the data for this paper. We have clarified that these were initial discussions with staff (see lines 105-108).

**Methods**

**Reviewer 1 and Reviewer 2 both highlighted where some interviews were particularly short and questioned why the interviews were not as long as we would typically expect.**

We recognise the interview time varied, which was often due to limited staff availability during the height of the pandemic, for example staff taking time on their breaks to talk to us and having to quickly stop the interview to turn to work. We have added a clarification into the participants section to highlight this context (see lines 204-206), and have also added more about this within the limitations section of the discussion (see lines 658-662).

**Reviewer 1 requested “more detail on what the six-phase structure of analysis consisted of”.**

We have added an additional section providing more detail about the six-phase structure of Thematic Analysis set out by Braun and Clarke (see lines 227-233).

**Reviewer 2 requested clarification as to why nine transcripts were selected separately.**

We conducted a familiarisation phase on all transcripts prior to in-depth analysis. We then selected nine transcripts due to them providing in-depth, rich data for initial coding and theme creation. After initial in-depth analysis on these nine transcripts, we applied the coding to the remaining transcripts. We have now clarified this in the manuscript (see lines 197-199).

**Reviewer 2 highlighted that “14 minutes is very short for an interview. Did you manage to get through all the questions?”**

We were able to get through all the priority questions marked in the interview schedule in the 14 minute interview as we knew time may be short with participants. We have added clarification in the methods section to show that we had priority questions in the interview schedule in case our participants did not have much time available for the interview (see lines 212-213).

**Reviewer 2 requested clarification when we stated that interview responses were triangulated against survey data.**

We have removed the section where we suggest that our findings were triangulated against survey data. Although we do reference a published article from this project, we had originally aimed to publish more of the quantitative data prior to this publication to therefore triangulate the qualitative findings to the quantitative survey data. However, as this is not yet available, we are unable to refer to other survey data at this time and have removed this section for clarity.

**Analysis**

**Reviewer 2 asked for the first sentence of the analysis to be reworded as the sentence was quite difficult to process.**

We have reworked this sentence to improve clarity and readability (see lines 257 - 258).

**Reviewer 2 requested format changes to the quotes, and that we include extract numbers.**

We have removed the quote marks and italics on the quotes to improve accessibility and have now included extract numbers. This can be seen throughout the analysis section of the manuscript. Thank you for raising this so that we could improve the accessibility of the article.

**Reviewer 2 stated that “the first extract (RE, hospital B) says the leader has been present, but I can’t see any praise there as the first sentence of commentary states”.**

We recognise that the praise is not clear from the extract itself and required further context. The response from RE regarding presence was following a question regarding their attitudes towards line management, and whether it was a feeling of togetherness or separation with their managers. In response, RE mentioned that they found no change from pre-covid, including their leader's presence and a lack of reluctance to get stuck in, therefore praising the presence of leadership. We have added this clarification into the manuscript (see lines 274-275). We have also reframed this section of the analysis to highlight the positive appraisal from leaders being present and engaging in the same tasks as frontline staff (see lines 277-281).

**Reviewer 2 highlighted how our arguments were less about shared social identity, but antecedents and consequences to shared social identity, and how our analysis required some reframing to address this.**

We thank reviewer 2 for their insight and have quite extensively reframed the analysis accordingly. Please find responses to specific comments below:

**Reviewer 2 highlighted that the quote on p11 (extract 2) that they “can see this could be explained by the interviewee seeing the manager as ingroup, but that kind of claim doesn’t seem to be in the extract itself. A bit more evidence is needed here. The commentary para ends with a reference to the literature on trust and prototypicality but again the quote doesn’t refer to trust”**

We have reframed this section of the analysis, where we now highlight that leaders’ presence and engagement in the same tasks as frontline staff provides antecedents to feeling part of the same group as leadership because these actions signal leaders and staff being equal or being prototypical (see lines 283-284, line 295, and lines 298-300).

**Reviewer 2 highlighted that the analysis of extract 3 that our statement that “non-present leadership who are seen to lack a shared understanding with staff could limit the ability for staff to adhere to COVID-19 safety guidance” was an inference, where “it would be better to demonstrate this relationship in the data rather than infer it (such inferences would be better in the Discussion where the implications of the research can be drawn out)”**

We have removed the inference of leadership limiting the ability for staff to adhere and have kept this just in the discussion section.

**Reviewer 2 highlighted that they would like “to see more direct evidence of interviewees categorising people as ingroup or outgroup, either by asking the interviewee (e.g., ‘did you feel part of the same team’, ‘did you feel unity with them?’) or spontaneous references to being part of the same group or ‘we’-talk”**

We have reframed this section of the analysis to highlight that the non-presence of leadership is seen to leave leaders unable to understand the challenges being faced by frontline staff, where this lack of understanding from leadership creates a separation between frontline staff and leadership, in turn contributing to categorising these non-present leaders as ‘other’. We have also now highlighted that the non-presence of leadership and lack of understanding of frontline challenges creates a lack of relational connection between leaders and staff, which demonstrates a lack of antecedents to shared social identity through leadership distance (see lines 308-322). We have also added a section within our limitations highlighting that we did rely on spontaneous mentions of group or team identity, where we allowed staff to create their own definition of who they considered part of their group/team given that this is dependent on the ward and staff structure (see lines 679-684 for limitations section, and lines 218-220 for discussion of staff crafting their own definitions of who was part of their group/team given variability in ward and staff structures).

**Reviewer 2 highlighted for extract 5 that “this extract talks about lack of shared experience being a problem but the commentary states that this then leads to no shared group membership. I can’t see the latter in the extract”**

We have reframed the analysis to highlight instead that the lack of shared experience renders the leaders in the office block as ‘other’ to frontline staff on the ward, where prior literature demonstrates the role of shared experiences in recognising others as part of the same social group (see lines 357-360).

**Reviewer 2 highlighted for extract 6 that our statement “Further, by highlighting that distant leadership lacked this shared experience, AE continues to categorise distant, non-present, and non-shared experience leaders as outgroup” was “a reasonable inference but it’s not in the data”.**

We have reframed this section of the analysis to instead highlight that AE’s comments demonstrate how antecedents to feeling like part of the same group as leadership are missing when these leaders are not present and do not share the same experiences as frontline staff (see lines 381-386).

**Discussion**

**Reviewer 1 highlighted the finding that “being physically co-present with staff was important to perceptions of effective leadership” which is a “relevant finding as the world moves to more hybrid models of working” and requested for this to “be highlighted a bit more in discussion, particularly acknowledging literature on relational and embodied approaches to leadership which seem salient for future research to better understand this phenomena”.**

We don’t feel we have enough evidence related to embodied approaches to leadership to discuss this in depth. However, we have added further discussion of the importance of considering the role of physical presence on effective leadership. We have discussed the importance of presence on creating an understanding of staff’s environment, experiences, and challenges faced in their workplace. We have highlighted that this is not to suggest that hybrid models of working are necessarily negative, but that strategy development is required to ensure leadership are able to develop this understanding of the staff environment/experience (see lines 620-628).

**Reviewer 1 highlighted “the finding on the need for a mediator with on-ground experience was also interesting”, noting that this was “perhaps an argument for a more distributed approach to leadership which could be highlighted and discussed”.**

We have highlighted within the practical implications section that a more distributed approach to leadership could be adopted, i.e., where those providing guidance and instruction to frontline staff could ensure their presence and engagement in order to facilitate both positive attitudes towards these leaders but to also promote positive attitudes towards the guidance they are providing (see lines 614 -617).

We have also highlighted the importance of leaders engaging with staff in order to understand the situation and barriers they are facing to build positive relations with staff to achieve solutions to these (see lines 602-605).

**Reviewer 1 highlighted that “there also seems to be a connection between approachability and care/caring leadership, which could be highlighted as an area for further exploration”**

We have added a discussion of how leaders being seen to care for staff was raised, which was appraised positively by staff and related to staff adherence to safety guidance. We have highlighted that this was mentioned only a few times, where we need future research to further explore the implications of caring leadership on the opportunities for leaders to (a) understand and (b) address the needs of staff (see lines 645-650).

**Reviewer 1 requested that we provide an explanation for why we felt the interviews tended to be brief, and “offer some reflections in the concluding sections of the paper on the limitations of the approach adopted”**

We have included an explanation for why some interviews were brief, which was primarily due to NHS staff availability being short and precious during COVID-19, where greater staff availability with staff would have provided more opportunity for in-depth discussions about staff relationships with leadership (see lines 658-662).

**Reviewer 1 recommended “some further critical reflection on the conceptual framework. To me, the issues discussed at the end of the paper reveal that leadership is a shared process whereby leadership can come from the front lines as well as from on high – it is relational. In other words, adopting an alternative view of leadership would help to develop insight that leadership in crisis conditions needs to be re-envisaged in relational terms”**

We have added further reflection and highlight now that the relational processes of presence and approachability were key to effective leadership and occurred regardless of formal leadership position (see lines 587-593).
